# Complete Genome Sequence of *Weissella cibaria* NH9449 and Comprehensive Comparative-Genomic Analysis: Genomic Diversity and Versatility Trait Revealed

**DOI:** 10.3389/fmicb.2022.826683

**Published:** 2022-05-19

**Authors:** Komwit Surachat, Duangporn Kantachote, Monwadee Wonglapsuwan, Arnon Chukamnerd, Panchalika Deachamag, Pimonsri Mittraparp-arthorn, Kongpop Jeenkeawpiam

**Affiliations:** ^1^Division of Computational Science, Faculty of Science, Prince of Songkla University, Songkhla, Thailand; ^2^Molecular Evolution and Computational Biology Research Unit, Faculty of Science, Prince of Songkla University, Songkhla, Thailand; ^3^Division of Biological Science, Faculty of Science, Prince of Songkla University, Songkhla, Thailand; ^4^Department of Biomedical Sciences and Biomedical Engineering, Faculty of Medicine, Prince of Songkla University, Songkhla, Thailand

**Keywords:** *Weissella cibaria*, pan-genome, phylogenetic analysis, lactic acid bacteria, probiotic

## Abstract

Lactic acid bacteria (LAB) in the genus *Weissella* spp. contain traits in their genome that confer versatility. In particular, *Weissella cibaria* encodes several beneficial genes that are useful in biotechnological applications. The complete genome of *W. cibaria* NH9449 was sequenced and an *in silico* comparative analysis was performed to gain insight into the genomic diversity among members of the genus *Weissella*. A total of 219 *Weissella* genomes were used in a bioinformatics analysis of pan-genomes, phylogenetics, self-defense mechanisms, virulence factors, antimicrobial resistance, and carbohydrate-active enzymes. These investigations showed that the strain NH9449 encodes several restriction-modification-related genes and a CRISPR-Cas region in its genome. The identification of carbohydrate-active enzyme-encoding genes indicated that this strain could be beneficial in biotechnological applications. The comparative genomic analysis reveals the very high genomic diversity in this genus, and some marked differences in genetic variation and genes among *Weissella* species. The calculated average amino acid identity (AAI) and phylogenetic analysis of core and accessory genes shows the possible existence of three new species in this genus. These new genomic insights into *Weissella* species and their biological functions could be useful in the food industry and other applications.

## Introduction

*Weissella*, a genus of Gram-positive lactic acid bacteria (LAB), is classified as a member of the Leuconostocaceae family (Fusco et al., 2015). LAB are catalase-negative, non-endospore-forming coccoid or rod-shaped bacteria. They prosper in a wide range of environments from fermented food to soil, feces, saliva, breast milk, urine, and banana leaves (Bjorkroth et al., 2002; Fairfax et al., 2014; Fusco et al., 2015), but most *Weissella* spp. that have been studied were isolated from sources associated with fermented food, e.g., *W. sagaensis* (Li Y. Q. et al., 2020), *W. hellenica* (Panthee et al., 2019), *W. confusa* (Falasconi et al., 2020), and *W. cibaria* (Shin et al., 2020). In other studies, *Weissella* spp. have shown various functions that could be suitable for biotechnological and medical applications. They could inhibit the growth of other bacteria (Yeu et al., 2021), act as starter cultures (Fusco et al., 2015; Yeong et al., 2020) and as probiotics (Huang et al., 2020; Kang et al., 2020) and prevent carcinogenic processes (Kwak et al., 2014).

With the rapid development of next-generation sequencing technology, more genomes from different *Weissella* species have been sequenced to identify genetic elements, study their potential functions, and compare phylogenetic relationships with other organisms. Several research works have focused on the potential of phenotypic and genotypic traits of *Weissella* spp. (Panthee et al., 2019; Shin et al., 2020; Yeong et al., 2020; Yuan et al., 2021). In particular *W. cibaria*, this species is a key member of the genus and exhibits a variety of traits and abilities. For instance, *W. cibaria* can produce bacteriocins such as weissellicin 110, which has strong activity against Gram-positive bacteria (Srionnual et al., 2007; Wu et al., 2015). Some *W. cibaria* strains were reported to encode and harbor several genes with useful biotechnological properties (Carlosama Adriana et al., 2021; Tenea and Hurtado, 2021). They also play an important role in heterofermentative metabolism and CO_2_ production from carbohydrate metabolism, which makes them suitable for use in fermentation processes (Ricciardi et al., 2009; Kang et al., 2016). Even though many genomic studies of several members of the genus have been carried out, the understanding of versatility traits, genomic diversity, and the complex phylogenetic relationships among species of this genus remains incomplete. Therefore, continued comparative genomic study of the genus is needed to fill these knowledge gaps.

In this work, we sequenced the complete genome of *W. cibaria* NH9449 isolated from Thai fermented pork sausage and performed a comprehensive comparative genomic analysis with other *Weissella* species. Functional annotation was performed to predict the presence of carbohydrate-active enzymes and bacteriocin-encoding genes and highlight traits in the fermentation activity of these bacterial strains. In addition to the *in silico* analysis, we produced an overview of the biological evolution of this genus to provide a more precise picture of phylogenetic relationships among *Weissella* spp. To our knowledge, this is the first meta-analysis to use all the whole-genome sequences of the genus *Weissella* in a comparative genomic analysis. Our results provide important information about the genomic diversity among species of this genus and a versatility trait that could be useful in the food fermentation industry.

## Materials and Methods

### Bacterial Strain and DNA Extraction

*Weissella cibaria* NH9449 was isolated from a traditional Thai-style fermented pork sausage at the Faculty of Science, Prince of Songkla University, Thailand. A single colony of *W. cibaria* NH9449 was cultivated in MRS broth at 37°C for 24 h under static conditions. Genomic DNA was purified and extracted using a DNeasy extraction kit (QIAGEN, Hilden, Germany) following the manufacturer’s instructions.

### Library Preparation and Whole-Genome Sequencing

We constructed two genome sequencing libraries to sequence both short and long reads using the Illumina and PacBio platforms, respectively. The purified genomic DNA was sent to be sequenced with a PacBio RSII sequencer (Pacific Biosciences, Menlo Park, CA, United States) by Macrogen (Seoul, South Korea). The sequencing library was prepared by 10 kb-protocol and P4-C2 chemistry to run in one single-molecule real-time (SMRT) cell. A total of 141,639 raw reads was obtained from the PacBio sequencer, with a total yield of 780 Mbp. The subread N50 and average subread lengths were 8,641 and 5,512, respectively. In addition, we sequenced the short-read library at Prince of Songkla University using a NextSeq 550 sequencer (Illumina, Inc., San Diego, CA, United States). Short-read sequencing was constructed with an insert size of 350 bp, using a Nextera XT DNA Library Prep Kit to generate 150-bp paired-end reads. The sequencer generated a total of 1 Gbp of paired-end reads. The Phred quality scores of the short reads reached Q30 for approximately 96% of all reads.

### Genome Assembly and Plasmid Identification

We first cleaned the raw data. Low-quality long-read sequences of less than 1,000 bp were filtered out using Canu v2.2 (Koren et al., 2017). Using fastp v0.23.0 (Chen et al., 2018), the short-read sequences were trimmed to improve the overall quality of all reads. The assembly process was then performed by Unicycler v0.4.7 (Wick et al., 2017) using both the short-read and long-read sequences. We then scanned for a small plasmid that could be absent from the assembly as described previously (Surachat et al., 2021a,b). Briefly, we mapped short-read sequences to draft assembly and extracted all unmapped reads to perform *de novo* assembly with spades v3.12 (Bankevich et al., 2012). Finally, we selected high coverage contigs only to check the similarity with data in the NR database of The National Center for Biotechnology Information (NCBI) server. Assembly polishing was performed by the Unicycler pipeline since it integrates the Pilon pipeline (Walker et al., 2014) to correct the draft assembly using short-read sequences. Finally, the quality and genome completeness of the assembly was assessed using Quast v4.0 (Gurevich et al., 2013) and Busco v5.2.2 (Seppey et al., 2019), respectively.

To confirm the presence of small plasmids, plasmid isolation was performed according to a published protocol (Yao et al., 2019) with some modifications. Briefly, the bacterial strain was grown under anaerobic conditions in MRS broth for 20 h at 37°C. A culture sample of 1 ml was placed in a sterile tube and centrifuged at 11,000 × *g* for 1 min. The pellets were resuspended in 250 μl of solution P1 (supplemented with 100 mg/ml lysozyme) of the QIAprep Spin Miniprep Kit (QIAGEN, Germany), and incubated for 10 min at 37°C. To completely remove lysozyme, the suspension was centrifuged at 11,000 × *g* for 1 min at room temperature. The pellets were washed twice by resuspension in 500 μl of 5% glucose solution and centrifuged at 11,000 × *g* for 1 min. The pellets were subjected to QIAprep Spin Miniprep Kit. Plasmid DNA was evaluated by agarose gel electrophoresis.

### Genome Annotation and Visualization

Genome annotations were predicted using an offline version of the NCBI Prokaryotic Genome Annotation Pipeline (PGAP) (Tatusova et al., 2016). Functional annotation to assign Clusters of Orthologous Groups (COG) was performed using the WebMGA web service (Wu et al., 2011) on the COG database (Tatusov et al., 2003). In addition, the clustered regularly interspaced short palindromic repeats (CRISPRs) were identified by CRISPRFinder (Grissa et al., 2007). Finally, the visualization of the genomic content was performed by the CGView Server BETA (Stothard et al., 2019).

### Comparative Genomic and Pan-Genome Analysis

The genomic sequences of all *Weissella* strains used in this study were downloaded from the NCBI server. We used FastANI v1.32 (Jain et al., 2018) to compute pairwise average nucleotide identity (ANI) among all genomes and the EzAAI pipeline (Kim et al., 2021) to calculate average amino acid identity (AAI) using all protein sequences of each genome. Core, accessory, and unique genes were identified by a pan-genome analysis performed with Roary v3.11.2 (Page et al., 2015), using a threshold of 70% BLASTp and default parameters. Core and accessory genes were identified as single nucleotide polymorphisms (SNPs) using SNP-sites (Page et al., 2016). Phylogenetic trees were constructed with the neighbor-joining method using RaxMLHPC (Stamatakis, 2006) and phylogenetic tree reliability was evaluated by the bootstrap method using 1000 replications. Tree visualization was illustrated in MEGA-X v10.1.7 (Kumar et al., 2018).

### Detection of Bacteriocin-Encoding Genes, Antimicrobial Resistance Genes, Plasmids, and Virulence-Associated Genes

To obtain all the bacteriocin-encoding genes of each bacterial strain, the BAGEL database (Van Heel et al., 2018) was searched using BLASTx with a cut-off *E*-value of 1e-10. The AMR genes and predicted phenotype/drug resistance profile of each genome were scanned with staramr v0.7.2^1^, which integrates several AMR gene-specific tools including ResFinder (Bortolaia et al., 2020) and PlasmidFinder (Carattoli and Hasman, 2020). Virulence factors were identified by searching the bacterial strains against the full dataset of the virulence factor database (VFDB) (Liu et al., 2019) with a cut-off *E*-value of 1e-30 and minimum identity of >70%.

### Prediction of CRISPR-Cas, R-M Systems, and Carbohydrate-Active Enzyme

CRISPR-Cas system was identified by CRISPRFinder (Grissa et al., 2007). In addition, Restriction-Modification (R-M) systems were explored using BLASTp analysis against the Rebase database (Roberts et al., 2015), with a setting of 70% identity threshold and a cut-off *E*-value of 1e-30. In addition, we identified the carbohydrate-active enzyme (CAZymes) (Lombard et al., 2014) families of all bacterial strains using run_dbcan^2^, a standalone tool of dbCAN2 (Zhang et al., 2018), with standard parameters.

## Results and Discussion

### *Weissella cibaria* NH9449 Genomic Features

**FIGURE 1:**
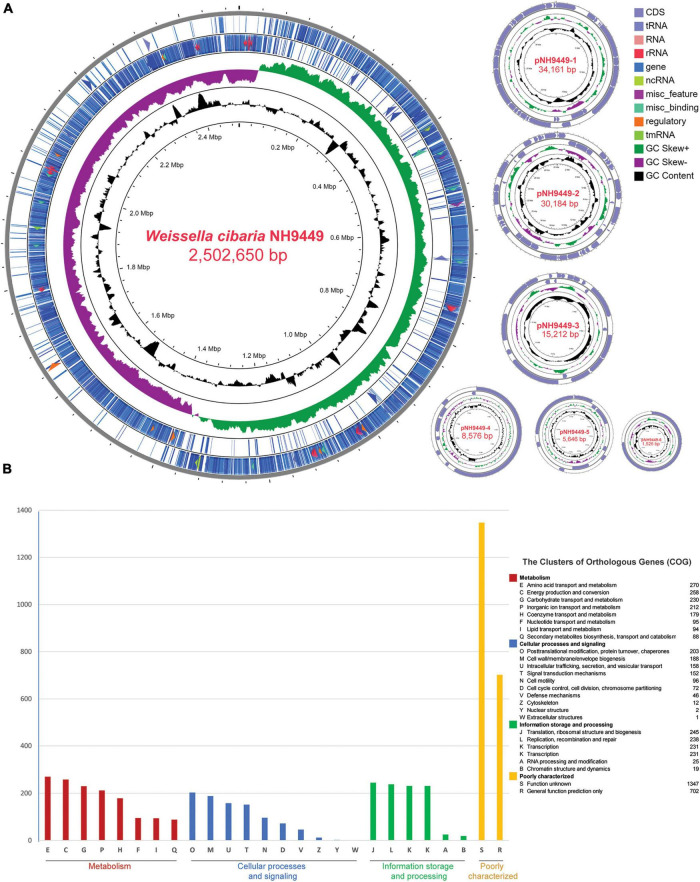
Genome visualization and annotation of *W. cibari*a NH9449. **(A)** Circular genome representation of chromosome and six plasmids. The element color of each circle is indicated in the legend. The following rings provide this information: forward strand CDS, reverse strand CDS, GC Skew, CG content, respectively. tRNA, RNA, rRNA, ncRNA, regulatory, and tmRNA are also located at the same ring of CDS based on their strand. **(B)** Clusters of Orthologous Groups (COG) identification of *W. cibari*a NH9449 genome.

The genome of *W. cibaria* NH9449 consists of a circular chromosome and six plasmids (Figure 1A). The genome size is 2.6 Mb. We identified a total of 2,390 protein-coding sequences, 28 rRNA genes, and 90 tRNA genes in this genome. The general genomic features are given in Table 1. The functional annotation predicted by the COG database is given in Figure 1B. In the NH9449 genome, more COGs (1490) were assigned to the metabolism functional category than any other category. Information storage and processing, and cellular processes and signaling were represented by 989 and 930 COGs, respectively. We found six plasmids in this genome; the size of the biggest plasmid was 34,161 bp and the smallest was only 1,526 bp. The presence of this small plasmid was confirmed using PCR amplification as shown in Supplementary Figure 1. The sequence similarities of the plasmids were close to various other strains of LAB species, e.g., *Lactiplantibacillus plantarum* (formerly *Lactobacillus plantarum*), *Leuconostoc mesenteroides*, *W. confusa*, and *W. soli*. Besides the NH9449 genome, we also identified seven and six plasmids in the genomes of *W. cibaria* CH2, and *W. paramesenteroides* WpK4, respectively. These findings could indicate that the NH9449 strain may have evolved to survive in new environments by acquiring new plasmids from closely related bacteria.

**TABLE 1:** Genome features of *W. cibaria* NH9449.

Feature	Chromosome	Plasmid					
		pNH9449-1	pNH9449-2	pNH9449-3	pNH9449-4	pNH9449-5	pNH9449-6
Size	2,502,650	64,161	30,184	15,212	8,576	5,646	1,526
GC content (%)	44.9	38.4	37.0	41.3	40.7	38.2	32.2
Protein coding genes	2,304	31	35	12	3	4	1
rRNA	28	–	–	–	–	–	–
tRNA	90	–	–	–	–	–	–
ncRNA	2	–	–	–	–	–	–
tmRNA	1	–	–	–	–	–	–

### Comparative Genomic Analysis of *Weissella* Species

**FIGURE 2:**
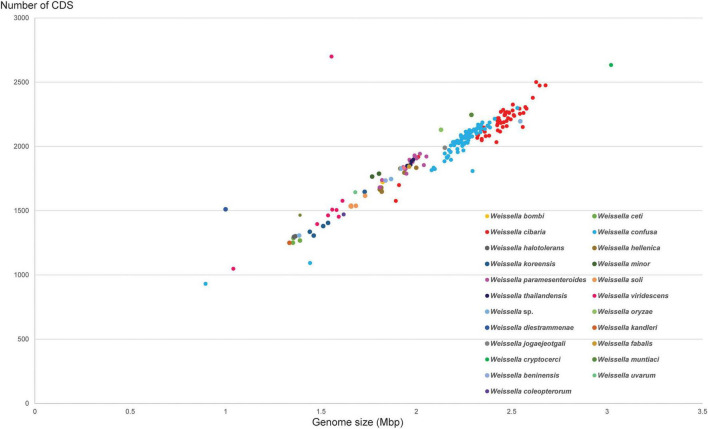
The genome size distribution was plotted against the number of CDSs identified in each bacterial strain among *Weissella* spp.

To better understand the genomic diversity among all *Weissella* species, we performed a comparative genome analysis against all 236 genomes in the NCBI database (Supplementary Table 1). After we had removed duplicated assembly names in different versions and genomes derived from metagenome assembly, the total number of genome sequences was 218. This total included 35 complete genomes, 43 scaffold levels, and 140 contig levels (Accessed:18 Oct 2021). The numbers of sequenced genomes of different *Weissella* spp. are shown in Supplementary Figure 2. The genome size distribution among the genus is quite wide, varying from 1.04 to 3.02 Mbp, while the GC content is between 35.3 and 45.4%. The average genome size of all strains was 2.14 Mbp and the number of identified CDSs was 1947. The scatterplot between genome size and the number of CDSs of *Weissella* species is shown in Figure 2. The size distribution tends to be consistent among the same species; the biggest genome was that of *Weissella cryptocerci* 26KH-42^T^ (Heo et al., 2019), the smallest that of *Weissella viridescens* MFPC16A2805 (Poirier et al., 2018). Naturally, genome size tends to increase as bacteria acquire mobile genetic components, transposons, and plasmids from other species during evolution (Li and Du, 2014). The size variation among the species within *Weissella* could also imply how these bacteria have adapted to survive in different environments by losing genes or receiving new ones (Bobay and Ochman, 2017). However, the genome size might also reflect the completeness of the assembly. Contamination, removal of contigs, and sequencing technology can affect the quality and size of the assembly.

**FIGURE 3:**
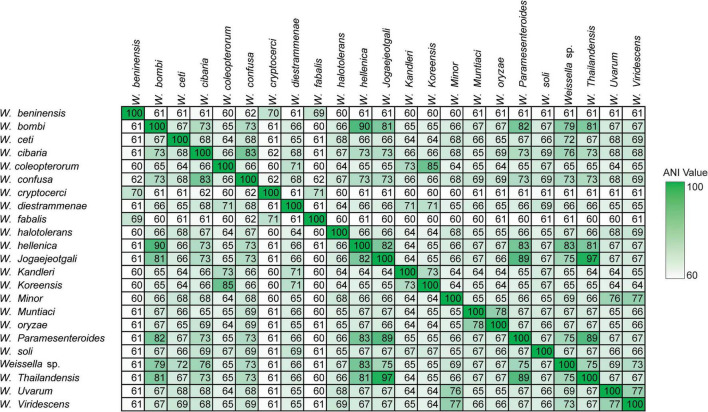
Interspecies similarities among the genus *Weissella*.

Furthermore, we computed and generated heatmaps of ANI values (Supplementary Figure 3) and AAI values (Supplementary Figure 4 and Supplementary Table 2) of 219 *Weissella* strains (including NH9449). Generally, taxonomic classification in the NCBI server is determined by ANI values (Ciufo et al., 2018). If the ANI value of a species compared to a reference strain is more than 95%, the species can be assigned as the same species as the reference in the taxonomic classification. As a result, some bacterial strains contained ANI values lower than 70%, we then analyzed the data based on AAI instead. The *W. cibaria* NH9449 strain shared sequence similarities from 96.25 to 99.23% with other *W. cibaria* strains. The average intraspecies similarity of all species is given in Table 2. Notably, we found several bacterial strains in *W. ceti* (1), *W. hellenica* (3), *W. paramesenteroides* (2), *Weissella* sp. (3), and *W. viridescens* (1), that presented AAI values lower than 95% among their groups, suggesting that species reclassification might be needed to reflect the updated data more accurately. For example, the shared AAI between *Weissella* sp. DD23 and *W. cibaria* ranged from 95.77 to 99.26%, suggesting this strain could be classified as *W. cibaria*. One of the findings of this analysis was that the AAI values between *W. ceti* CECT 7719 and all other *W. ceti* strains ranges from 60.59 to 85.0%. This has a certain relevance to the classification of these species since the taxonomic status of all *W. ceti* strains in the NCBI server, except CECT 7719, is “Inconclusive,” which means the ANI/AAI values of these strains are lower than 95%. Since *W. ceti* CECT 7719 was announced as a new species in 2011 (Vela et al., 2011), we suggest based on this analysis, that the other *W. ceti* strains, including NC36 (Ladner et al., 2013), WS08 (Figueiredo et al., 2014b), WS74, and WS105 (Figueiredo et al., 2014a), might also be new species and should be further investigated. In addition, analysis of the average AAI showed that interspecies similarities are between 59.81 and 97.05% (Figure 3). We found that *W. jogaejeotgali* and *W. thailandensis*, in agreement with a previous report (Panthee et al., 2019), present very high average similarity values over 97%. However, *W. jogaejeotgali* was reclassified as a heterotypic synonym of *W. thailandensis* in 2019 (Kwak et al., 2019). This analysis revealed that at least 11 of the studied strains presented AAI values lower than the criterion for taxonomic classification (95%). This finding could lead to the discovery of at least three new species/subspecies in this genus.

**TABLE 2:** Average amino acid identity information among *Weissella* spp.

Species	No. of genomes	Minimum AAI (%)	No. of genome AAI < 95%
*W. beninensis*	1	100.00	0
*W. bombi*	2	99.99	0
*W. ceti*	5	84.78	1
*W. cibaria*	60	95.37	0
*W. confusa*	76	96.38	0
*W. coleopterorum*	1	100.00	0
*W. cryptocerci*	1	100.00	0
*W. diestrammenae*	1	100.00	0
*W. fabalis*	1	100.00	0
*W. halotolerans*	2	98.76	0
*W. hellenica*	8	93.52	4
*W. jogaejeotgali*	1	100.00	0
*W. kandleri*	1	100.00	0
*W. koreensis*	5	99.26	0
*W. minor*	2	96.81	0
*W. muntiaci*	1	100.00	0
*W. oryzae*	1	100.00	0
*W. paramesenteroides*	27	81.44	2
*W. soli*	5	99.13	0
*Weissella sp.*	5	66.15	3
*W. thailandensis*	4	99.99	0
*W. uvarum*	1	100.00	0
*W. viridescens*	8	91.56	1

**FIGURE 4:**
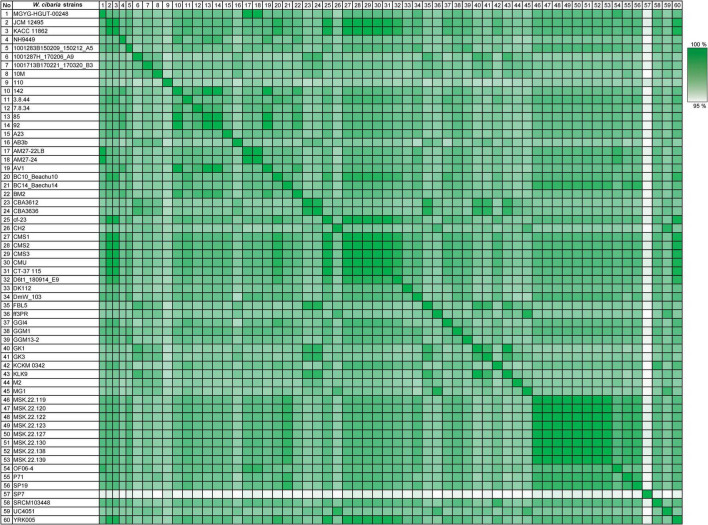
Average amino acid identity analysis of 60 *W. cibaria* strains. The numbers display the percentage similarity between *W. cibaria* strains, where the colors vary from white (low similarity) to green (high similarity).

Also, AAI similarity was identified among only 60 *W. cibaria* strains (Figure 4). The similarities among these *W. cibaria* strains ranged from 96.08 to 100%. Every strain showed consistent similarities which were all over 95%. The bacterial strains isolated from similar sources seemed to share closer AAI values than strains isolated from different sources. For instance, the species with the AAI values farthest from and closest to NH9449 were *W. cibaria* SP7 isolated from cow feces, and *W. cibaria* AV1 isolated from fermented food, respectively. Habitat differences have a direct influence on which genetic elements are acquired or lost through horizontal gene transfer (HGT) to and from the environment. This process affects genetic variation and diversity in the same species (Wiedenbeck and Cohan, 2011; Lee et al., 2022).

### Pan-Genome and Phylogenetic Analysis

**FIGURE 5:**
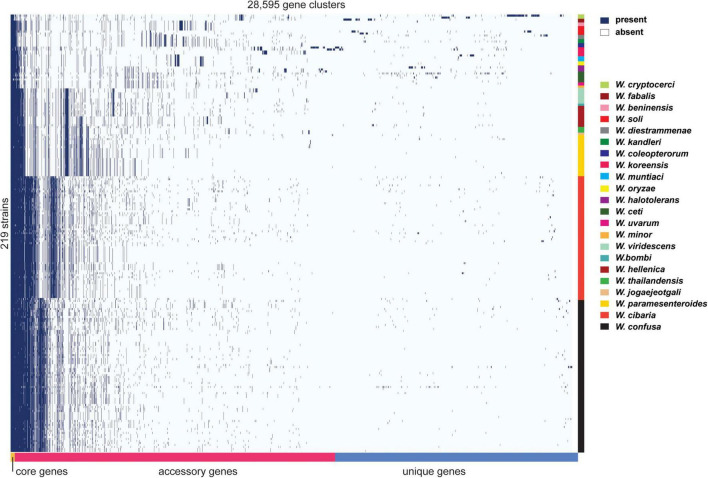
Pan-genome matrix of 219 *Weissella* genomes from 22 species. The right-hand color bars indicate the species boundary in the matrix. Lime, cherry, rose, red, gray, emerald, dark blue, pink, blue, yellow, violet, basil, magenta, cream, mint, teal, apple, green, wood, gold, orange, and black colors (from top to bottom) represent *W. cryptocerci*, *W. fabalis*, *W. beninensis*, *W. soli*, *W. diestrammenae*, *W. kandleri*, *W. coleopterorum*, *W. koreensis*, *W. muntiaci*, *W. oryzae*, *W. halotolerans*, *W. ceti*, *W. uvarum*, *W. minor*, *W. viridescens*, *W. bombi*, *W. hellenica*, *W. thailandensis*, *W. jogaejeotgali*, *W. paramesenteroides*, *W. cibaria*, and *W. confusa*, respectively.

The diversity of the genus *Weissella* was clear from the pan-genome analysis. The analysis clustered genes from all genomes of interest into subgroups, namely core genes (found in >99%), soft core genes (found in 95–99%), shell genes (found in 15–95%), and cloud genes (found in <15%). The number of core genes, soft core genes, shell genes and cloud genes identified at the genus level were 165, 151, 3,055, and 25,224, respectively. It is clear that *W. cibaria*, *W. confusa*, *W. hellenica*, *W. paramesenteroides*, *W. jogaejeotgali* and *W. thailandensis* tend to share more core genes if other species are removed from the analysis (Figure 5). In contrast, other *Weissella* species, e.g., *W. oryzae*, *W. cryptocerci*, and *W. fabalis*, carried several unique genes that spread over the top of matrix, producing a large portion of cloud genes. Those genes were mostly predicted as hypothetical proteins while the annotated genes were diverse and could be found in several organisms. For example, many genes commonly found in *Bacillus subtilis*, *Escherichia coli*, *Lactococcus lactis* and other bacteria, were also identified among the cloud genes, e.g., *adhR*, *mhqA*, *rhaS*, and *lcnD*. These gene families might be acquired from microorganisms encountered by *Weissella* spp. during their evolution in a diverse range of ecological niches.

**FIGURE 6:**
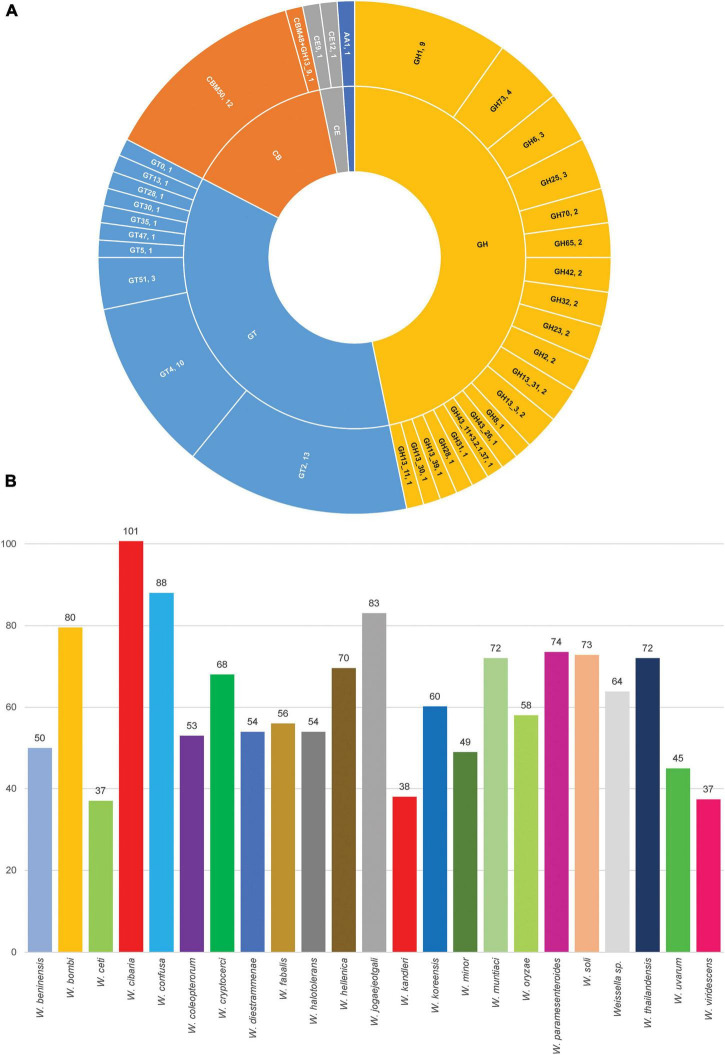
Carbohydrate-active enzyme analysis. **(A)** CAZymes distribution in *W. cibari*a NH9449. **(B)** Average count of CAZymes distribution in *Weissella* spp.

At the species level, *W. cibaria* had 1,187 core genes, 344 soft core genes, 1,403 shell genes and 3,880 cloud genes. In addition, the presence/absence matrix showed a big portion of homologous genes that were present in the cluster containing *W. cibaria* and *W. confusa*. These two species, which were the closest among all the species, shared 747 core genes and 146 soft core genes. Ongoing HGT and gene loss may have helped one of the two evolve from the other (Polz et al., 2013). Unsurprisingly, we found that 40, 30, and 10% of core genes of *W. cibaria* and *W. confusa* were, respectively, assigned into the “Metabolism,” “Information storage and processing,” “Cellular processes and signaling” categories of COG classification. Most core genes were classified in several subcategories, e.g., “Translation, ribosomal structure and biogenesis (J)”, “Amino acid transport and metabolism (E),” and “Carbohydrate transport and metabolism (G).” The three COG categories accessory genes were most commonly assigned to were “Cell wall/membrane/envelope biogenesis (M),” “Replication, recombination and repair (L),” and “Carbohydrate transport and metabolism (G).” We found that core genes and accessory genes both contained a high abundance of genes involved in carbohydrate transport and metabolism. We then further identified those genes using the CAZymes database to explore more related functions in *Weissella* spp. As a result, we found that *W. cibaria* NH9449 encodes a total of 105 CAZyme domain sequences in five families, including Glycoside Hydrolase (GH), Glycosyl Transferase (GT), Carbohydrate Esterase (CE), Auxiliary Activity (AA), and Carbohydrate-Binding Module (CBM) (Figure 6A). The highest frequency of CAZyme predicted in the NH9449 genome was found in the GH family (43) followed by the GT family (33). This investigation could indicate the ability of this bacterial strain to metabolize carbohydrate, which could be beneficial in several biotechnological applications. We investigated this characteristic among all members of the genus *Weissella*, by searching all strains against the CAZyme database. *W. cibaria* 85 and *W. viridescens* NCTC13645 presented the highest and the lowest number of CAZyme domain sequences of 112, and 21, respectively. The average numbers of CAZyme genes identified in each *Weissella* species are presented in Figure 6B. *W. cibaria* (101), *W. confusa* (88), and *W. jogaejeotgali* (83) are the three species that encode most CAZyme genes in their genomes. Our identification results are consistent with those of a previous study (Yuan et al., 2021), which found that *W. confusa* and *W. cibaria* metabolize carbohydrates and could be beneficial in several applications. These findings could also imply that most *Weissella* species, and especially *W. cibaria* and *W. confusa*, specialize in carbohydrate metabolism, which helps control mass degradation in the fermentation process.

The openness of the pan-genome of the genus *Weissella* was apparent from the pan-genome analysis (Supplementary Figure 5A). When more genomes were included in the analysis, the pan-genome plots of *W. cibaria* strains tended to be closed (Supplementary Figure 5B). Therefore, sequencing more *W. cibaria* strains might not be useful since the chance of discovering new genetic elements is probably slim. However, *W. cibaria* was not the only species whose pan-genome showed a tendency to be closed. In a previous report (Yuan et al., 2021), the pan-genome of 39 *W. confusa* strains was also open but in this study, when we analyzed 77 strains of *W. confusa*, the pan-genome status tended to be closed. In addition, we observed that the core genome sizes of *W. cibaria* strains seemed to be stable after sequencing around 50 genomes including ≈ 1,200 genes (Supplementary Figure 5B). At the genus level, the core genome plot of *Weissella* reached the stable stage after sampling around 150 genomes at ≈ 165 genes (Supplementary Figure 5A). However, when more strains were added, the core genome size of the genus remains very small while the pan-genome size increases. We suggest that sequencing other *Weissella* species would be more likely to reveal new gene families and there are many genomes of other species that have not yet been fully investigated.

**FIGURE 7:**
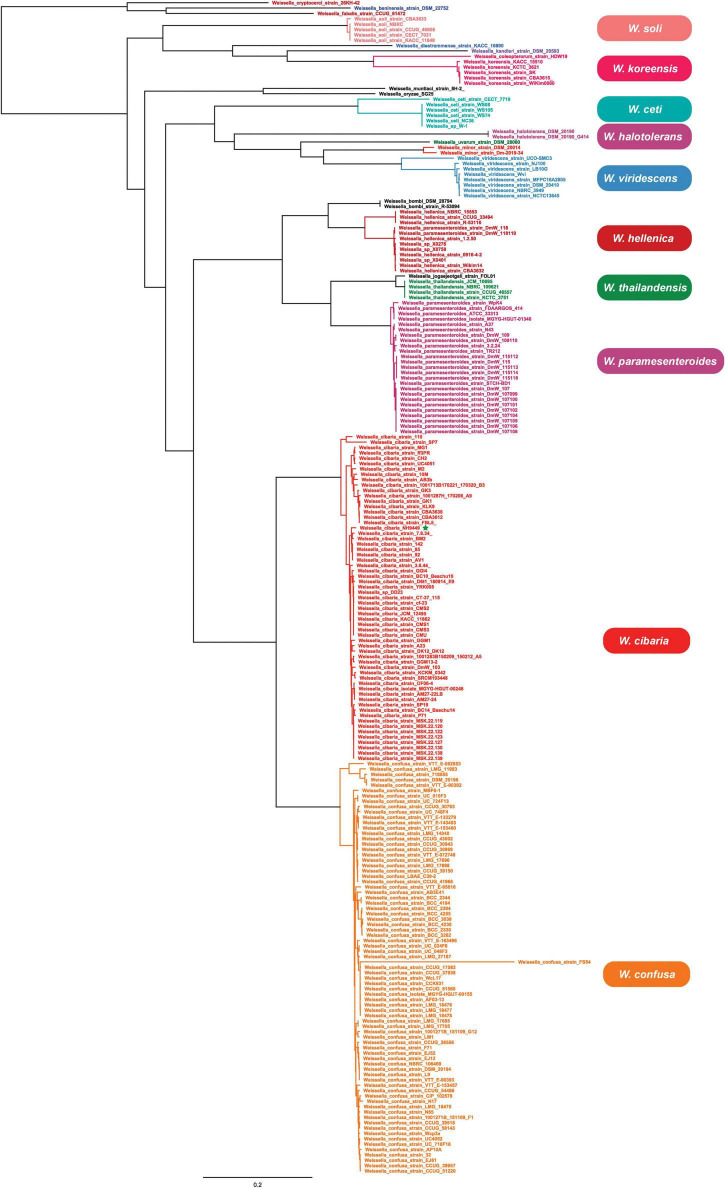
The phylogenetic tree is based on the core genes of 219 *Weissella* genomes.

The phylogenetic analyses were constructed based on core genes (Figure 7) and accessory genes (Supplementary Figure 6) of all *Weissella* strains. The NH9449 was grouped in the same clade as *W. cibaria* strains 7.8.34, BM2, 142, 85, 92, and AV1, respectively, implying that these bacterial strains shared close genetic components since they were all isolated from the same ecological niche. Furthermore, both phylogenetic trees formed a distinct clade for each *Weissella* species, enabling their taxonomic classification in this genus. We found that some bacterial strains were misplaced in the trees. This finding was consistent with our AAI analysis. Notably, *W. hellenica*, *W. viridescens*, and *W. ceti* branches were divided into two distinct clades and showed AAI similarities below the species criterion (95%) (Table 3). *W. hellenica* formed two distinct groups in both phylogenetic trees, based on core genes and accessory genes. *W. hellenica* CCUG 33494 (representative strain) grouped with strains NBRC 15553 and R-53116. In contrast, in the other clade, *W. hellenica* strains Wikim14, CBA3632, 0916-4-2, and 1.2.50 grouped in the same branch together with *W. paramesenteroides* strains DmW_118 and DmW_118119, and *Weissella* sp. X0278, X0401, and X0750. Since the similarity of 16S rRNA sequences of *Weissella* spp. are very close, previous taxonomic classifications could have been directly affected. So, we suggest that these two *W. paramesenteroides* strains and three *Weissella* sp. could be reclassified as *W. hellenica*. Based on our AAI calculations and proposed phylogenetic tree analysis, we suggest that the findings of this analysis might lead to the discovery of new species/subspecies in the genus *Weissella*. Also, using only the 16S rRNA marker might not be enough to classify species in this genus, and more markers could be considered for a better resolution in the taxonomic classification.

**TABLE 3:** NCBI taxonomy status and AAI values.

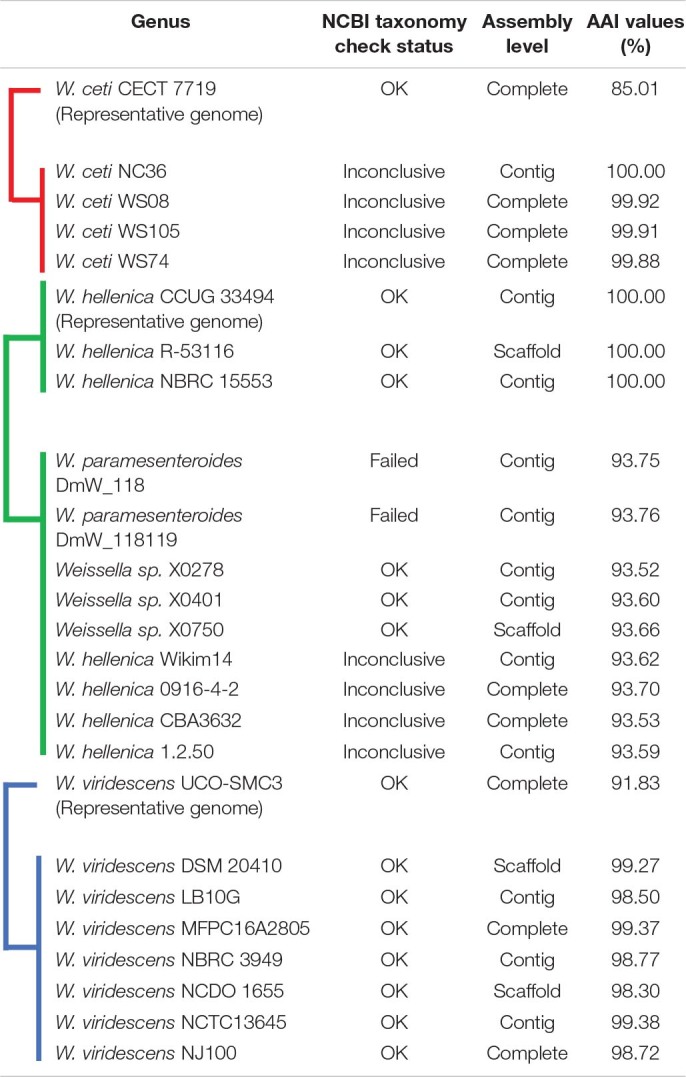

### Bacteriocin Diversity in *Weissella* spp.

Bacteriocins are ribosomally synthesized antibacterial peptides/proteins produced by bacteria to inhibit the growth of other bacterial strains (De Vuyst and Leroy, 2007). Since LAB contain several bacteriocin-encoding genes in many bacterial strains (Riley and Wertz, 2002), they have been deployed to perform important functions in microbiology applications. Therefore, we looked to identify the bacteriocin-encoding genes in all *Weissella* strains based on a BLASTx analysis against the bacteriocin database and the BAGEL server. We found no bacteriocin-encoding gene in the NH9449 genome and that other *Weissella* spp. encoded bacteriocin-encoding genes only in classes I and III. In class I, we found only two strains that contained bacteriocin-encoding genes. These strains were *W. cryptocerci* 26KH-42 (nukacinISK-1 and lactococcinDR) and *W. cibaria* 1001713B170221_170320_B3 (cytolysin_ClyLs). We did not identify any class II bacteriocin in any strain. Surprisingly, weisselin A (Papagianni and Papamichael, 2012), weissellicin M and Y (Masuda et al., 2012), which were originally *W. paramesenteroides* DX and *W. hellenica* QU13, were not found in any *Weissella* strain. In class III, eight strains from three species, including *W. thailandensis* (4), *W. soli* (3), and *W. minor* (1), were found to encode large protein bacteriocins (Table 4). In addition, weissellicin 110 (Srionnual et al., 2007), which was not included in the BAGEL database, was identified in only four strains of *W. cibaria*. This analysis clarified the frequency of bacteriocin-encoding genes in *Weissella* spp. We found that only a few bacterial strains in the genus encode ribosomally synthesized antibacterial peptides, while LAB in other genera encode a larger diversity of bacteriocin-encoding genes: for example, *Pediococcus* (Jiang et al., 2020; Surachat et al., 2021b), *Lactobacillus* (Kaskoniene et al., 2017; Surachat et al., 2021a), and *Enterococcus* (Nes et al., 2007; Lopez-Cuellar et al., 2016). Even though bacteriocins are a crucial biological tool for inhibiting pathogens and other microorganisms, most *Weissella* spp. use genomic traits such as CRISPR and R-M systems to maintain their stability.

**TABLE 4:** Bacteriocin-encoding genes identified in *Weissella* spp.

	Bacterial strains	Bacteriocin
Class I	*W. cryptocerci* 26KH-42	LactococcinDR
		NukacinISK-1
	*W. cibaria* 1001713B170221_170320_B3	Cytolysin_ClyLs
	*W. minor* Dm-2019-34	Zoocin_A
		Bacteriocin_helveticin_J
		Helveticin-J
		Helveticin
Class III	*W. soli* CCUG 46608	Closticin_574
	*W. soli* CECT_7031	
	*W. soli* NBRC 106074	
	*W. thailandensis* CCUG_46557	Zoocin_A
	*W. thailandensis* NBRC 109621	
	*W. thailandensis* KCTC_3751	
	*W. thailandensis* JCM_10695	
–	*W. cibaria 110*	
	*W. cibaria* BC10	Weissellicin 110
	*W. cibaria* DK12	
	*W. cibaria* GGI4	

### CRISPR-Cas and R-M Systems

We explored CRISPR-Cas regions and R-M systems in all bacterial strains used in this study (Supplementary Figure 7 and Supplementary Table 3). From the CRISPR-Cas prediction, we found that 94.5 and 68.5% of all strains, respectively, contained the CRISPR array and the cas locus in their genome. Most of the cas loci were detected with CRISPR arrays, while some of them were detected without CRISPR arrays. Overall, type I-E, II-A, III-A, and III-C CRISPR-Cas systems were identified in 2, 36, 1, and 4 *Weissella* genomes, respectively, whereas we could not identify the subtype of type I CRISPR-Cas system in most of the *Weissella* genomes (*n* = 160). The combinations of type I and II-A (9.6%), type I and III-C (1.8%), and type I-E, II-A, and III-A (0.5%) CRISPR-Cas systems were also identified in these *Weissella* genomes. A CRISPR region at 2,231,486 to 2,231,580 (94 bp) with a DR length of 23 bp was found in the NH9449 genome. For R-M prediction, we found that 76.3, 66.0, 30.6, and 26.3% of all strains contained type I, II, III, and IV R-M systems, respectively. Three types of R-M systems, I, II, and III, were predicted in *W. cibaria* NH9449. We did not find any type V R-M system in any strain in this analysis. This finding might indicate that these *Weissella* genomes have been infected with bacteriophages. Furthermore, it could imply that the presence of CRISPR-Cas and R-M regions in most *Weissella* genomes probably provides adaptive immunity against foreign genetic elements from bacteriophages or extracellular plasmids (Abriouel et al., 2017; Xing et al., 2017).

### Antimicrobial Resistance and Virulence Factors in the Genus *Weissella*

To assess antimicrobial resistance (AMR), we investigated the presence of AMR genes in all 219 *Weissella* strains. An AMR gene was found only in *W. cibaria* 142 and *W. cibaria* AV1, which, respectively, encoded the *bla*_TEM–1A_ and *bla*_TEM–162_ genes. These 2 β-lactamase genes may provide resistance to ampicillin. Thus, we propose that contamination with *bla*_TEM_-carrying strains should be a concern in food production. Additionally, we still need to clarify how these AMR genes are acquired as well as the location of the genes. If AMR genes are located on mobile genetic elements (such as plasmids), they can be transferred to other *Weissella* spp., especially pathogenic species. This study identified the ColRNAI plasmid in *W. cibaria* 92. The ColRNAI plasmid is predominantly found in *Klebsiella pneumoniae*, especially multidrug- and carbapenem-resistant strains (Dong et al., 2018; Agyepong et al., 2019; Yu et al., 2019; Li R. C. et al., 2020) and has also been detected in *Escherichia coli* and *Salmonella* spp. (Litrup et al., 2017; Hadziabdic et al., 2018). Therefore, the discovery of the ColRNAI plasmid in *W. cibaria* 92 implies acquisition from *K. pneumoniae*, *Escherichia coli*, or *Salmonella* spp. Previous studies have reported that the ColRNAI plasmid carries many AMR genes, including the carbapenem resistance gene (*bla*_KPC–2_) and colistin resistance gene (*mcr-3*) as well as other AMR genes (*bla*_CTX–M–55_, *aac(3)-IId*, *tet*(A), *qnrS1*, *sul2*, *catA2*, *floR*, *strA*) (Litrup et al., 2017; Hadziabdic et al., 2018; Agyepong et al., 2019; Yu et al., 2019; Shelenkov et al., 2020). Although no AMR gene was identified in this ColRNAI plasmid, we still need to be concerned about this ColRNAI-harboring strain. Since neither AMR genes nor plasmids were detected in *W. cibaria* NH9449 in our study, the NH9449 may not be harmful to humans and can perhaps be used in biotechnological applications.

Virulence-related factors in the *Weissella* genomes were identified based on a BLASTn search against the full dataset of virulence factors in the VFDB database. We found 27 matches of putative virulence factor genes in the NH9449 genome, including *tufA* (elongation factor Tu), *lisR* (two-component response regulator), *hasC* (DP-glucose pyrophosphorylase) and *SMU_322c* (glucose-1-phosphate uridylyltransferase). These genes were previously reported in *W. hellenica* 0916-4-2 (Panthee et al., 2019), and *W. cibaria* UTNGt21O (Tenea and Hurtado, 2021). *W. confusa* FS54 encodes 133 virulence-associated genes, which is the highest number in this genus. The average number of occurrences of virulence factors of all members was found to be only 23, which were almost the same putative virulence factor genes that we found in NH9449. These results show that most *Weissella* genomes encode common virulence-associated genes which have been widely identified in *Lactobacillus, Pediococcus*, *Enterococcus* and other LAB species (Muñoz-Atienza et al., 2013; Chajęcka-Wierzchowska et al., 2017; Colautti et al., 2022). However, further study should be conducted to explore the functions and characteristics of these virulence genes.

## Conclusion

The complete genome of *W. cibaria* NH9449 was sequenced and a comparative genomic analysis was performed against all members in the genus *Weissella*. We found several self-defense mechanisms in the NH9449 genome, including CRISPR-Cas and R-M systems. Also, the significantly higher abundance of CAZyme encoding genes in this bacterial strain compared to other *Weissella* strains indicates its greater ability to metabolize carbohydrate. The absence of virulence factors and AMR genes in the NH9449 genome imply that the strain could be safe for use in food processing. Moreover, the comparative genomic analysis revealed an extremely high genomic diversity among this genus and a high variation in the genome content. Besides *W. cibaria* NH9449, several other *Weissella* spp. did not exhibit AMR genes or harmful virulence factors and could possibly be considered as safe. Some strains produce antimicrobial substances that can inhibit a broad spectrum of pathogens, and we found that *Weissella* species, especially *W. cibaria* and *W. confusa*, carried several CAZyme genes. These properties could be useful for biotechnological applications, especially in food fermentation and probiotics. However, these bacterial strains have to be carefully selected and tested before use since *Weissella* spp. are still not Generally Recognized As Safe (GRAS). Furthermore, the results of AAI and phylogenetic analyses suggest that several *Weissella* spp. should be reclassified and that reclassification might uncover new species of *W. ceti*, *W. hellenica* and *W. viridescens*. However, further investigations are still needed to confirm this assumption. This study provides new information about *W. cibaria* NH9449 and the genomic diversity among *Weissella* spp. and could be a guideline for future studies.

## Data Availability Statement

The complete chromosome and six plasmids of *W. cibaria* NH9449 have been deposited at GenBank with accession numbers CP061503–CP061509. The BioProject and BioSample accession numbers are PRJNA663405 and SAMN16131644, respectively. The sequence raw reads of short reads and long reads were deposited in SRA with the respective accession numbers SRR18492148 and SRR18492149.

## Author Contributions

KS, DK, and MW designed the study. KS and PD performed the genome sequencing and annotation. KS and KJ performed the comparative genomic analysis. KS, DK, PM-A, AC, and MW wrote the manuscript. DK and KS integrated the research and critically revised the manuscript for important intellectual content. KS, DK, PM-A, and MW approved the final version of the manuscript. All authors contributed to the article and approved the submitted version.

## Conflict of Interest

The authors declare that the research was conducted in the absence of any commercial or financial relationships that could be construed as a potential conflict of interest.

## Publisher’s Note

All claims expressed in this article are solely those of the authors and do not necessarily represent those of their affiliated organizations, or those of the publisher, the editors and the reviewers. Any product that may be evaluated in this article, or claim that may be made by its manufacturer, is not guaranteed or endorsed by the publisher.

## Footnotes

1
https://github.com/phac-nml/staramr

2
https://github.com/linnabrown/run_dbcan

## Supplementary Material

Supplementary Figure 1 PCR amplification of plasmid size 1,526 bp.

Supplementary Figure 2 The chart shows the number of genomes of each *Weissella* sp. used in this study.

Supplementary Figure 3 In the results of the ANI analysis of 219 *Weissella* strains, similarity levels are represented by red, orange, yellow, lime, and green. Below and including 60% similarity is represented by red, 61 to 70% by orange, 71 to 80% by yellow, 81 to 90% by lime, and 91 to 100% by green.

Supplementary Figure 4 In the AAI analysis of 219 *Weissella* strains, similarity levels from low to high are represented by red, orange, yellow, lime, and green.

Supplementary Figure 5 The plots were produced from the pan-genome analysis at the genus **(A)** and species **(B)** level. The plots show the number of genes found when adding more genomes in the dataset of the genus and the dataset of *W. cibaria* strains only.

Supplementary Figure 6 The phylogenetic tree is based on accessory genes of 219 *Weissella* genomes.

Supplementary Figure 7 The gene presence/absence matrix of R-M system related genes, CRISPR-cas systems, and bacteriocins was produced from analysis of all the strains studied in this work.

Supplementary Table 1 List of bacterial strains used in this study.

Supplementary Table 2 AAI Analysis of 219 *Weissella* strains.

Supplementary Table 3 CRISPR identification in *Weissella* strains.
