# Supplementary figures and images for: Complete Genome Sequence of Weissella cibaria NH9449 and Comprehensive Comparative-Genomic Analysis: Genomic Diversity and Versatility Trait Revealed
Source: Front Microbiol. 2022 May 19;13:826683. doi: 10.3389/fmicb.2022.826683 (PMC9161744; doi:10.3389/fmicb.2022.826683)

bp

1

2

2000

1500

500

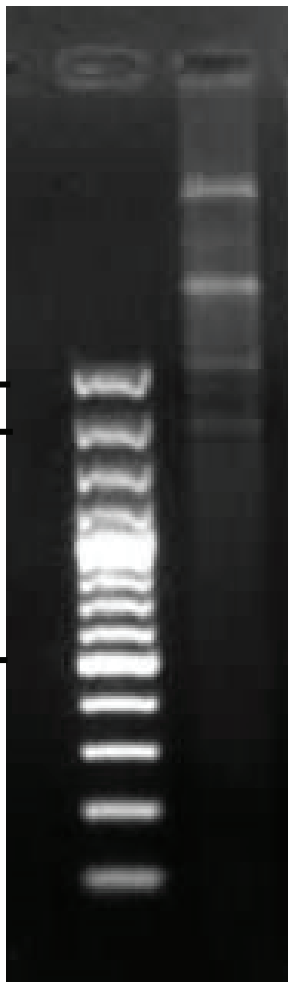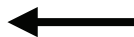

Supplement: Supplementary Figure 1 — PCR amplification of plasmid size 1,526 bp. [file Image_1.PDF]

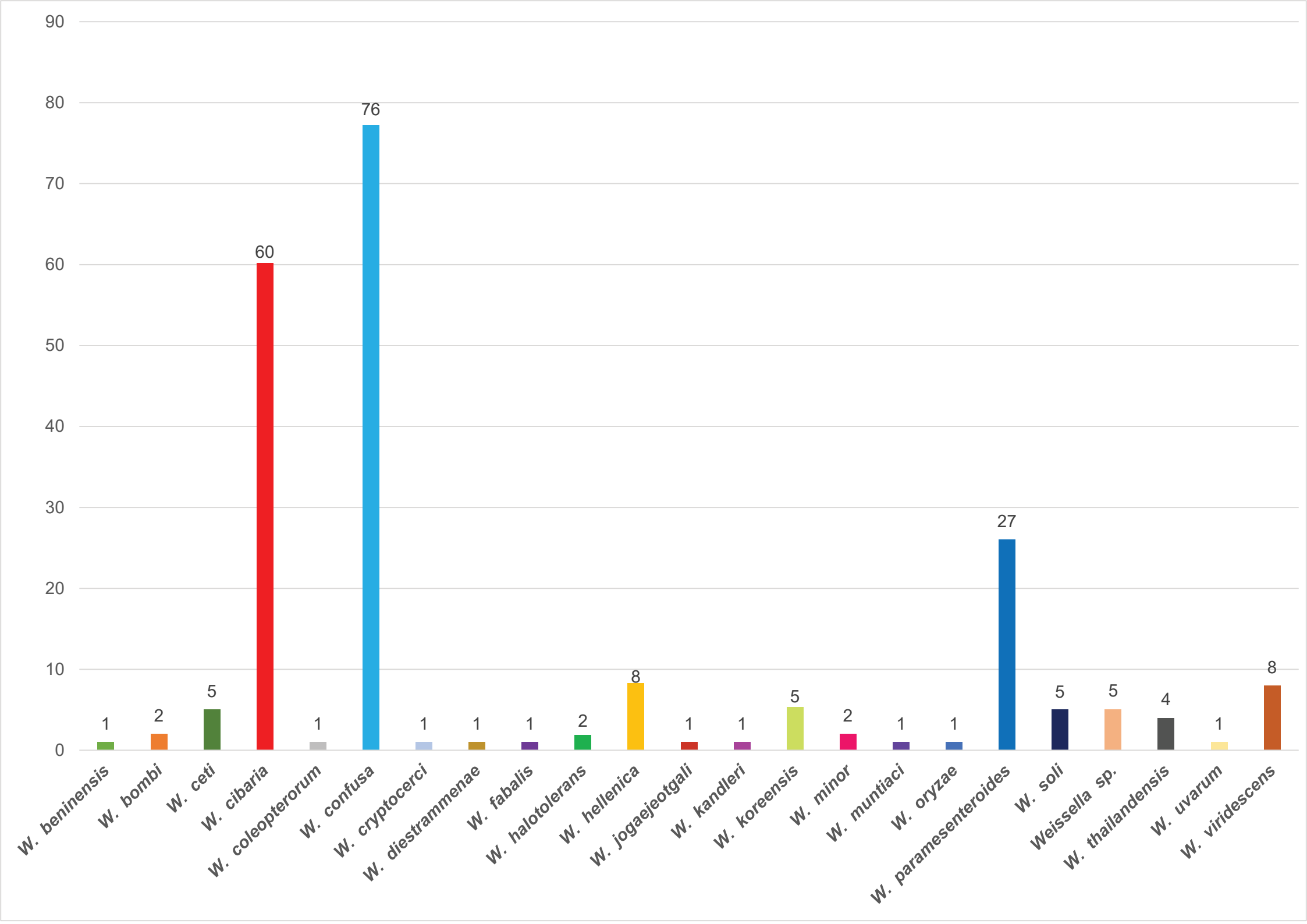

Supplement: Supplementary Figure 2 — The chart shows the number of genomes of each Weissella sp. used in this study. [file Image_2.PDF]

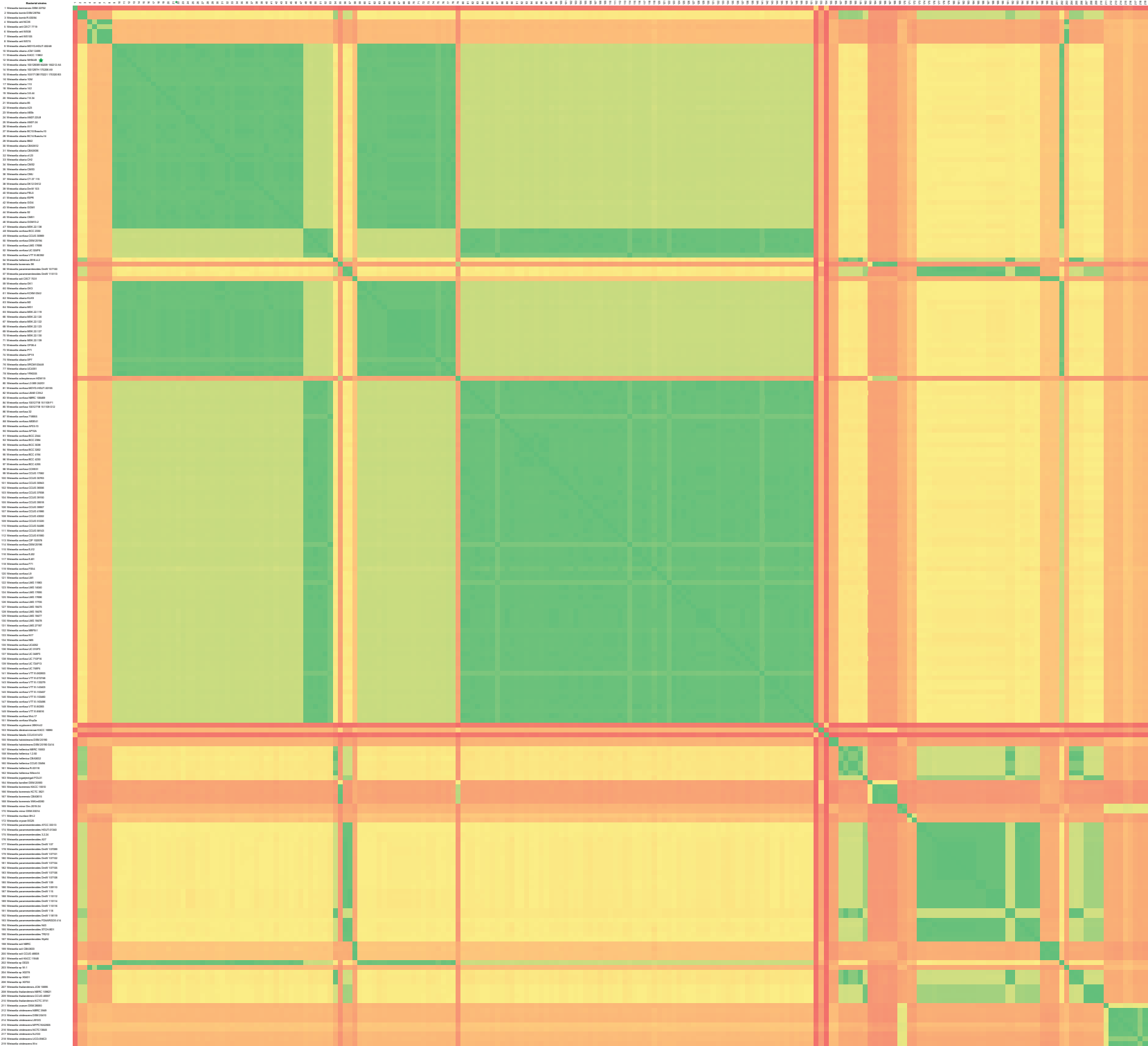

Supplement: Supplementary Figure 3 — In the results of the ANI analysis of 219 Weissella strains, similarity levels are represented by red, orange, yellow, lime, and green. Below and including 60% similarity is represented by red, 61 to 70% by orange, 71 to 80% by yellow, 81 to 90% by lime, and 91 to 100% by green. [file Image_3.PDF]

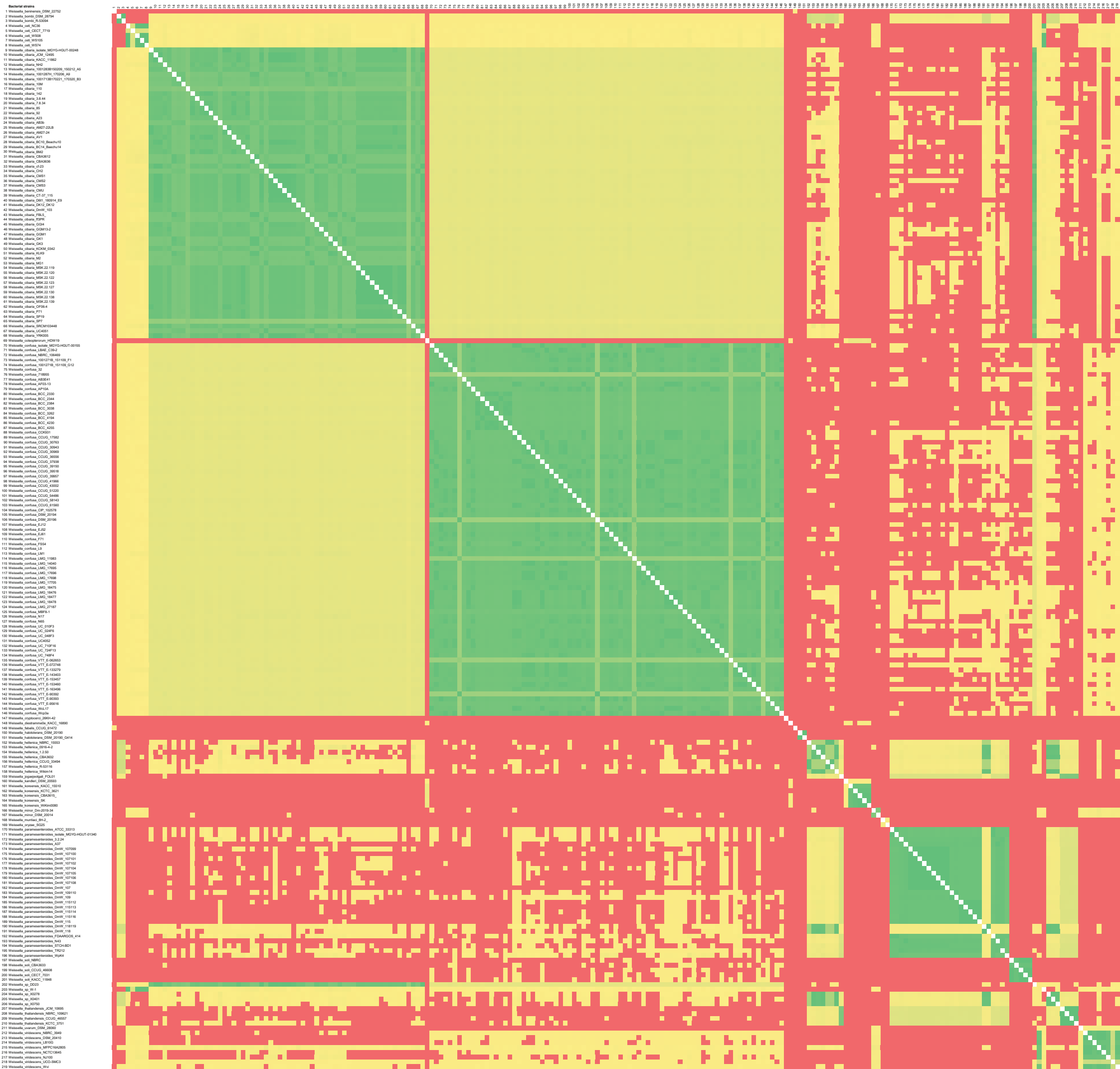

Supplement: Supplementary Figure 4 — In the AAI analysis of 219 Weissella strains, similarity levels from low to high are represented by red, orange, yellow, lime, and green. [file Image_4.PDF]

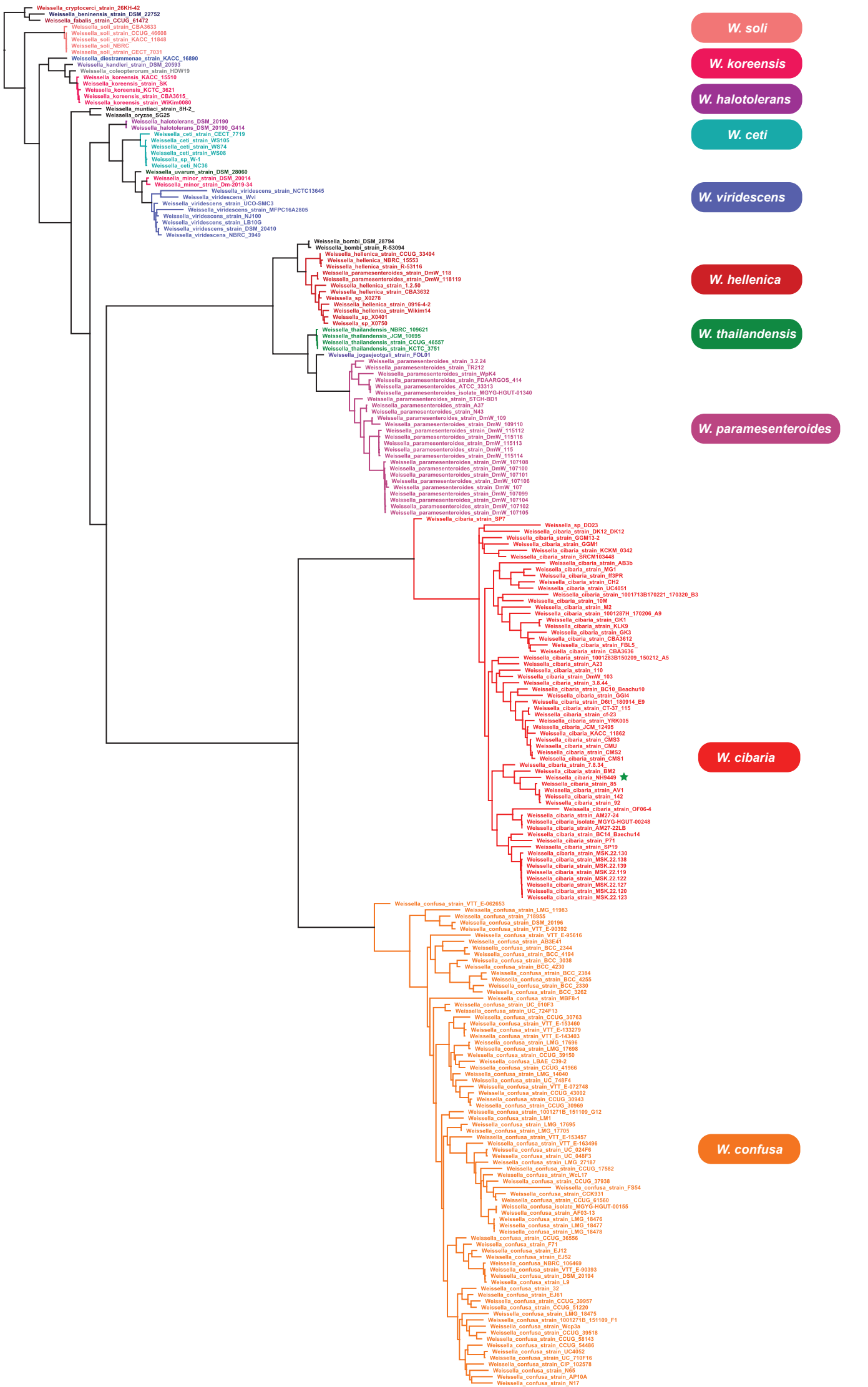

Supplement: Supplementary Figure 6 — The phylogenetic tree is based on accessory genes of 219 Weissella genomes. [file Image_6.PDF]
