# Supplementary material for: Complete Genome Sequence of Weissella cibaria NH9449 and Comprehensive Comparative-Genomic Analysis: Genomic Diversity and Versatility Trait Revealed
Source: Front Microbiol. 2022 May 19;13:826683. doi: 10.3389/fmicb.2022.826683 (PMC9161744; doi:10.3389/fmicb.2022.826683)
Supplement: Supplementary Figure 5 — The plots were produced from the pan-genome analysis at the genus (A) and species (B) level. The plots show the number of genes found when adding more genomes in the dataset of the genus and the dataset of W. cibaria strains only. [file Image_5.PDF]

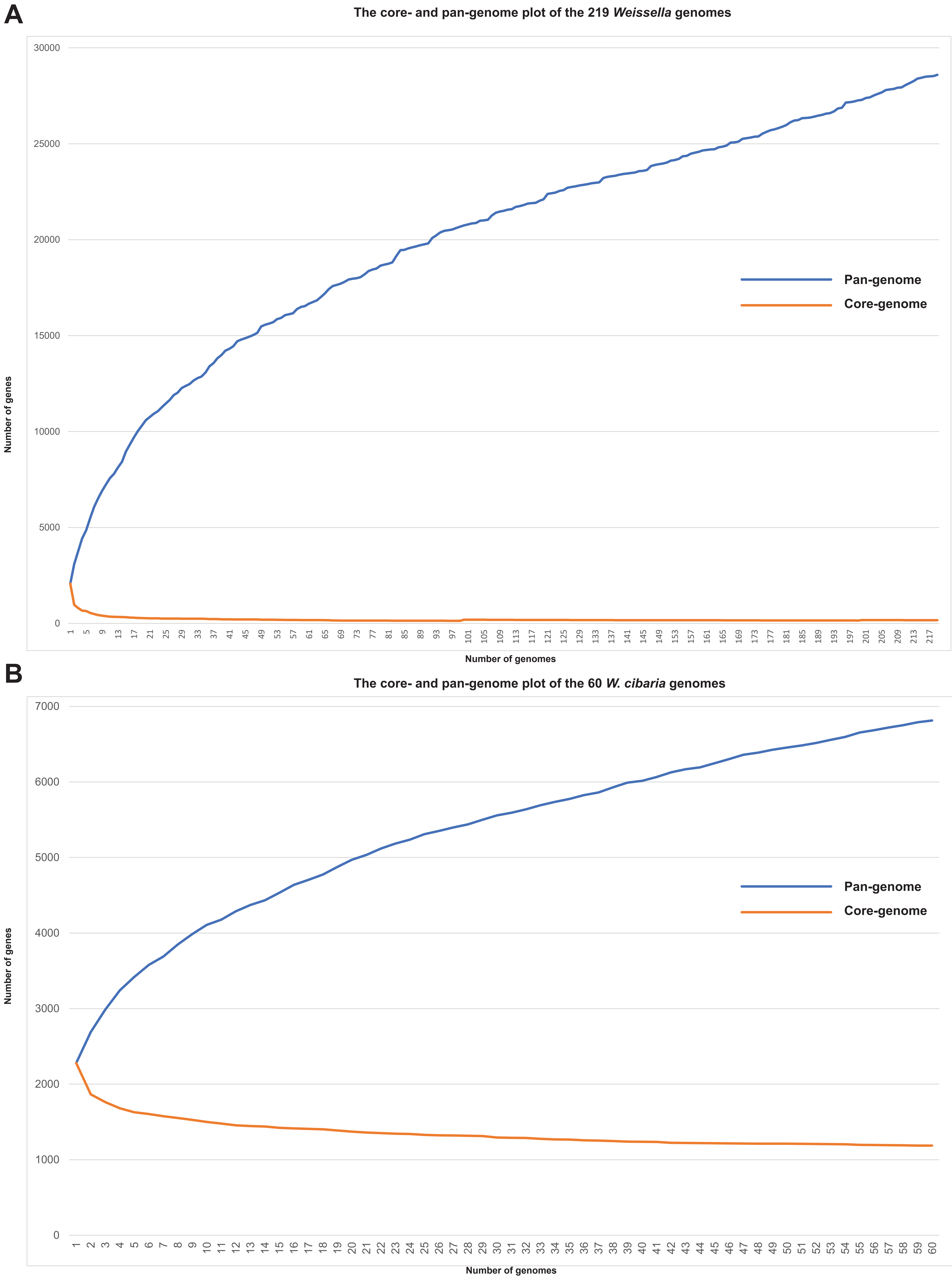
A
The core- and pan-genome plot of the 219 Weissella genomes
Number of genes
Number of genomes
Pan-genome
Core-genome
B
The core- and pan-genome plot of the 60 W. cibaria genomes
Number of genes
Number of genomes
Pan-genome
Core-genome
