# Supplementary material for: Complete Genome Sequence of Weissella cibaria NH9449 and Comprehensive Comparative-Genomic Analysis: Genomic Diversity and Versatility Trait Revealed
Source: Front Microbiol. 2022 May 19;13:826683. doi: 10.3389/fmicb.2022.826683 (PMC9161744; doi:10.3389/fmicb.2022.826683)
Supplement: Supplementary Figure 7 — The gene presence/absence matrix of R-M system related genes, CRISPR-cas systems, and bacteriocins was produced from analysis of all the strains studied in this work. [file Image_7.PDF]

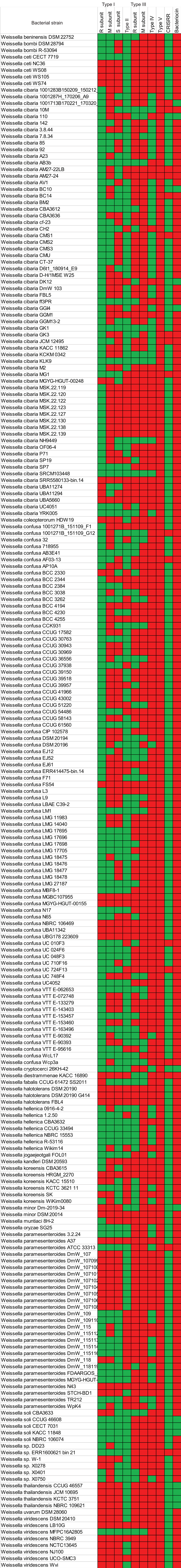
Type I
Type III
Bacterial strain
R subunit
M subunit
S subunit
Type II
R subunit
M subunit
Type IV
Type V
CRISRR
Bacteriocin
Weissella beninensis DSM 22752
Weissella bombi DSM 28794
Weissella bombi R-53094
Weissella ceti CECT 7719
Weissella ceti NC36
Weissella ceti WS08
Weissella ceti WS105
Weissella ceti WS74
Weissella cibaria 1001283B150209_150212_
Weissella cibaria 1001287H_170206_A9
Weissella cibaria 1001713B170221_170320_
Weissella cibaria 10M
Weissella cibaria 110
Weissella cibaria 142
Weissella cibaria 3.8.44
Weissella cibaria 7.8.34
Weissella cibaria 85
Weissella cibaria 92
Weissella cibaria A23
Weissella cibaria AB3b
Weissella cibaria AM27-22LB
Weissella cibaria AM27-24
Weissella cibaria AV1
Weissella cibaria BC10
Weissella cibaria BC14
Weissella cibaria BM2
Weissella cibaria CBA3612
Weissella cibaria CBA3636
Weissella cibaria cf-23
Weissella cibaria CH2
Weissella cibaria CMS1
Weissella cibaria CMS2
Weissella cibaria CMS3
Weissella cibaria CMU
Weissella cibaria CT-37
Weissella cibaria D6t1_180914_E9
Weissella cibaria D-H/1MSE W25
Weissella cibaria DK12
Weissella cibaria DmW 103
Weissella cibaria FBL5
Weissella cibaria ff3PR
Weissella cibaria GGI4
Weissella cibaria GGM1
Weissella cibaria GGM13-2
Weissella cibaria GK1
Weissella cibaria GK3
Weissella cibaria JCM 12495
Weissella cibaria KACC 11862
Weissella cibaria KCKM 0342
Weissella cibaria KLK9
Weissella cibaria M2
Weissella cibaria MG1
Weissella cibaria MGYG-HGUT-00248
Weissella cibaria MSK.22.119
Weissella cibaria MSK.22.120
Weissella cibaria MSK.22.122
Weissella cibaria MSK.22.123
Weissella cibaria MSK.22.127
Weissella cibaria MSK.22.130
Weissella cibaria MSK.22.138
Weissella cibaria MSK.22.139
Weissella cibaria NH9449
Weissella cibaria OF06-4
Weissella cibaria P71
Weissella cibaria SP19
Weissella cibaria SP7
Weissella cibaria SRCM103448
Weissella cibaria SRR5580133-bin.14
Weissella cibaria UBA11274
Weissella cibaria UBA11294
Weissella cibaria UBA5660
Weissella cibaria UC4051
Weissella cibaria YRK005
Weissella coleopterorum HDW19
Weissella confusa 1001271B_151109_F1
Weissella confusa 1001271B_151109_G12
Weissella confusa 32
Weissella confusa 718955
Weissella confusa AB3E41
Weissella confusa AF03-13
Weissella confusa AP10A
Weissella confusa BCC 2330
Weissella confusa BCC 2344
Weissella confusa BCC 2384
Weissella confusa BCC 3038
Weissella confusa BCC 3262
Weissella confusa BCC 4194
Weissella confusa BCC 4230
Weissella confusa BCC 4255
Weissella confusa CCK931
Weissella confusa CCUG 17582
Weissella confusa CCUG 30763
Weissella confusa CCUG 30943
Weissella confusa CCUG 30969
Weissella confusa CCUG 36556
Weissella confusa CCUG 37938
Weissella confusa CCUG 39150
Weissella confusa CCUG 39518
Weissella confusa CCUG 39957
Weissella confusa CCUG 41966
Weissella confusa CCUG 43002
Weissella confusa CCUG 51220
Weissella confusa CCUG 54486
Weissella confusa CCUG 58143
Weissella confusa CCUG 61560
Weissella confusa CIP 102578
Weissella confusa DSM 20194
Weissella confusa DSM 20196
Weissella confusa EJ12
Weissella confusa EJ52
Weissella confusa EJ61
Weissella confusa ERR414475-bin.14
Weissella confusa F71
Weissella confusa FS54
Weissella confusa L3
Weissella confusa L9
Weissella confusa LBAE C39-2
Weissella confusa LM1
Weissella confusa LMG 11983
Weissella confusa LMG 14040
Weissella confusa LMG 17695
Weissella confusa LMG 17696
Weissella confusa LMG 17698
Weissella confusa LMG 17705
Weissella confusa LMG 18475
Weissella confusa LMG 18476
Weissella confusa LMG 18477
Weissella confusa LMG 18478
Weissella confusa LMG 27187
Weissella confusa MBF8-1
Weissella confusa MGBC107955
Weissella confusa MGYG-HGUT-00155
Weissella confusa N17
Weissella confusa N65
Weissella confusa NBRC 106469
Weissella confusa UBA11342
Weissella confusa UBG178 223609
Weissella confusa UC 010F3
Weissella confusa UC 024F6
Weissella confusa UC 048F3
Weissella confusa UC 710F16
Weissella confusa UC 724F13
Weissella confusa UC 748F4
Weissella confusa UC4052
Weissella confusa VTT E-062653
Weissella confusa VTT E-072748
Weissella confusa VTT E-133279
Weissella confusa VTT E-143403
Weissella confusa VTT E-153457
Weissella confusa VTT E-153460
Weissella confusa VTT E-163496
Weissella confusa VTT E-90392
Weissella confusa VTT E-90393
Weissella confusa VTT E-95616
Weissella confusa WcL17
Weissella confusa Wcp3a
Weissella cryptocerci 26KH-42
Weissella diestrammenae KACC 16890
Weissella fabalis CCUG 61472 SS2011
Weissella halotolerans DSM 20190
Weissella halotolerans DSM 20190 G414
Weissella halotolerans FBL4
Weissella hellenica 0916-4-2
Weissella hellenica 1.2.50
Weissella hellenica CBA3632
Weissella hellenica CCUG 33494
Weissella hellenica NBRC 15553
Weissella hellenica R-53116
Weissella hellenica Wikim14
Weissella jogaejeotgali FOL01
Weissella kandleri DSM 20593
Weissella koreensis CBA3615
Weissella koreensis HRGM_2270
Weissella koreensis KACC 15510
Weissella koreensis KCTC 3621 11
Weissella koreensis SK
Weissella koreensis WiKim0080
Weissella minor Dm-2019-34
Weissella minor DSM 20014
Weissella muntiaci 8H-2
Weissella oryzae SG25
Weissella paramesenteroides 3.2.24
Weissella paramesenteroides A37
Weissella paramesenteroides ATCC 33313
Weissella paramesenteroides DmW_107
Weissella paramesenteroides DmW_10709
Weissella paramesenteroides DmW_10710
Weissella paramesenteroides DmW_10710
Weissella paramesenteroides DmW_10710
Weissella paramesenteroides DmW_10710
Weissella paramesenteroides DmW_10710
Weissella paramesenteroides DmW_10710
Weissella paramesenteroides DmW_10710
Weissella paramesenteroides DmW_109
Weissella paramesenteroides DmW_10911
Weissella paramesenteroides DmW_115
Weissella paramesenteroides DmW_11511
Weissella paramesenteroides DmW_11511
Weissella paramesenteroides DmW_11511
Weissella paramesenteroides DmW_11511
Weissella paramesenteroides DmW_118
Weissella paramesenteroides DmW_11811
Weissella paramesenteroides FDAARGOS_
Weissella paramesenteroides MGYG-HGUT-
Weissella paramesenteroides N43
Weissella paramesenteroides STCH-BD1
Weissella paramesenteroides TR212
Weissella paramesenteroides WpK4
Weissella soli CBA3633
Weissella soli CCUG 46608
Weissella soli CECT 7031
Weissella soli KACC 11848
Weissella soli NBRC 106074
Weissella sp. DD23
Weissella sp. ERR1600621 bin 21
Weissella sp. W-1
Weissella sp. X0278
Weissella sp. X0401
Weissella sp. X0750
Weissella thailandensis CCUG 46557
Weissella thailandensis JCM 10695
Weissella thailandensis KCTC 3751
Weissella thailandensis NBRC 109621
Weissella uvarum DSM 28060
Weissella viridescens DSM 20410
Weissella viridescens LB10G
Weissella viridescens MFPC16A2805
Weissella viridescens NBRC 3949
Weissella viridescens NCTC13645
Weissella viridescens NJ100
Weissella viridescens UCO-SMC3
Weissella viridescens Wvi
